# Efficacy and safety of oliceridine in daytime hysteroscopic polypectomy: a randomized, double-blind, single-center controlled trial

**DOI:** 10.3389/fmed.2026.1819353

**Published:** 2026-05-22

**Authors:** Yuan Wang, Yufeng Qiu, Lan Wang, Jiaming Kang, Wenbin Zhang, Pragyanshu Khare, Xinnan Song

**Affiliations:** 1 Department of Anesthesiology, The Fifth Clinical Medical College, Guilin Medical University, Guilin, China; 2 Department of Gynecology, The Fifth Clinical Medical College, Guilin Medical University, Guilin, China; 3 Department of Anesthesiology, Friedrich Alexander Universität Erlangen-Nürnberg, Erlangen, Germany; 4 Department of Pharmacy, Birla Institute of Technology and Science-Pilani, Pilani, Rajasthan, India

**Keywords:** clinical recovery scores, endometrial polyps, hysteroscopic polypectomy, intravenous general anesthesia, oliceridine

## Abstract

**Background:**

This study aimed to compare the effects of oliceridine, fentanyl, and sufentanil on early recovery and perioperative safety during hysteroscopic polypectomy.

**Methods:**

A single-center, double-blind, randomized controlled trial enrolled 92 patients aged 18–65 with ASA I–II and BMI 18–30 kg/m^2^ undergoing elective intravenous general anesthesia for the procedure. Patients were randomly assigned in a 1:1:1 ratio to the fentanyl (*n* = 30), sufentanil (*n* = 31), and oliceridine (*n* = 31) groups. The primary outcome was the Clinical Recovery Score (CRS) at 5, 10, and 15 min post-awakening. Secondary outcomes included time to eye opening on verbal command, time to full awakening, intraoperative vital signs, and adverse events.

**Results:**

The oliceridine group had significantly higher CRS at 5, 10, and 15 min after awakening than the fentanyl and sufentanil groups (overall *p* = 0.002, *p* = 0.009, and *p* < 0.001, respectively). The time to eye opening on verbal command was shorter in the oliceridine group than in the fentanyl and sufentanil groups (overall *p* < 0.001). The time to full awakening was also shorter in the oliceridine group than in the fentanyl and sufentanil groups (overall *p* < 0.001). At T2 (after anesthetic stabilization and before surgery), the oliceridine group had higher RR than the fentanyl and sufentanil groups (both *p* < 0.001), and higher SpO_2_ than the fentanyl group (*p* = 0.0178). No significant differences were observed among the groups in intraoperative motor response, assisted ventilation, or rescue analgesic use.

**Conclusion:**

For hysteroscopic polypectomy, propofol combined with oliceridine was associated with improved early recovery outcomes and more favorable respiratory-related observations compared with fentanyl and sufentanil. These findings support the potential value of oliceridine in facilitating earlier recovery in day surgery, although confirmation in larger studies with more comprehensive physiological monitoring is required.

## Introduction

Endometrial polyps represent a common benign gynecological disorder. They are characterized by localized hyperplasia of the endometrial glands and stroma, which results in the development of single or multiple polypoid structures protruding into the uterine cavity (1, 2). Hysteroscopic polypectomy is the preferred clinical treatment approach. As a day-surgery procedure, it should enable prompt patient discharge without compromising safety. Consequently, effective perioperative pain management and the optimization of recovery quality are of crucial importance (3).

Most traditional opioids exert their pharmacological effects by activating μ-opioid receptors, which subsequently trigger downstream G protein-coupled and β-arrestin2-mediated signaling pathways (4). Current research suggests that the G protein signaling pathway primarily mediates analgesic effects (5). In contrast, activation of the β-arrestin2 signaling pathway is closely associated with opioid-related adverse events (ORAEs), such as gastrointestinal dysfunction, opioid-induced respiratory depression (OIRD), and excessive sedation. ORAEs not only prolong the duration of surgery and anesthesia, thereby increasing medical costs, but also pose significant risks to patients undergoing gynecological surgery. For instance, vomiting may lead to bleeding or other complications (6).

Oliceridine is a novel G-protein-biased opioid receptor agonist. Its unique mechanism of action entails selectively activating the G-protein signaling pathway and minimizing the recruitment of β-arrestin2, which in turn reduces β-arrestin2-mediated ORAEs (7). Studies have demonstrated that oliceridine offers potential advantages in gastrointestinal endoscopy and postoperative pain management over traditional opioids (8). Due to these pharmacological effects, oliceridine has been proposed as a potential alternative opioid with a differentiated adverse-effect profile, although its clinical role remains to be further defined (7).

Although current research has presented encouraging clinical data, most of these studies have focused on comparisons with morphine. However, direct comparisons with commonly used fentanyl-based opioids in routine clinical practice remain limited (9). Moreover, the potential differences in early recovery profiles and perioperative respiratory characteristics among these agents have not been well defined, particularly in short-duration ambulatory procedures.

Therefore, the present study was designed to compare early post-anesthetic recovery and perioperative safety among oliceridine, fentanyl, and sufentanil in patients undergoing hysteroscopic polypectomy. The primary endpoint was the Clinical Recovery Score (CRS) assessed at 5, 10, and 15 min after awakening. Secondary endpoints included comparisons of time to eye opening on verbal command, time to full awakening, perioperative vital signs (HR, RR, MAP, and SpO_2_), and the incidence of intraoperative adverse events among the three groups.

## Methods

### Study design

This study was designed as a randomized, double-blind trial. The study was conducted between September 2025 and November 2025. Patients were randomized in a 1:1:1 ratio using a computer-generated sequence. Allocation concealment was ensured with opaque, sealed, sequentially numbered envelopes, which were opened sequentially after enrollment confirmation by an anesthesia nurse not involved in anesthesia management, postoperative assessment, or data analysis. The nurse prepared fentanyl (2 μg/kg), sufentanil (0.2 μg/kg), or oliceridine (40 μg/kg), and all study drugs were diluted to the same final volume of 5 ml in identical syringes. Patients, the attending anesthesiologist, and the surgical team were blinded to treatment allocation. CRS assessments were performed by the attending anesthesiologist, who remained unaware of group assignment, although no independent blinded assessor was used.

The entire anesthesia process was carried out by the same anesthesiologist, and the surgical procedure was performed by the same surgical team. Emergency unblinding was permitted only to determine rescue medications for critical adverse events or during other medical emergencies.

All patients were required to fast for 6 h and abstain from drinking for 4 h before surgery. Upon entering the operating room, intravenous access was established, and the following parameters were monitored: non-invasive blood pressure (NIBP), pulse oxygen saturation (SpO_2_), electrocardiography (ECG), and Bispectral Index (BIS). After being administered oxygen via a face mask for 3 min while in the supine position, three NIBP measurements were taken, and the average value was used as the patient's pre-anesthesia baseline.

The anesthesia method used was non-intubated general anesthesia. Patients were placed in the lithotomy position. Hysteroscopic polypectomy was performed using a rigid hysteroscope with a cold knife technique. Visualization was achieved using an endoscopic imaging system (model YS-398), and uterine distension was maintained with normal saline. All procedures were performed by the same surgical team to ensure procedural consistency. During the procedure, propofol injection solution was administered via intravenous infusion at a rate of 4–6 mg/kg/h. Surgery commenced when the eyelash reflex had disappeared and the BIS was maintained between 40 and 60. In the event of patient movement during surgery, an additional dose of 0.5 mg/kg of propofol was administered. After the surgery, the anesthesia nurse carried out data collection.

The primary endpoint was the CRS of patients at 5 min, 10 min, and 15 min post-awakening. CRS provides a structured assessment of immediate post-anesthetic recovery across key physiological and functional domains relevant to discharge readiness for day surgery patients, and is well aligned with the predefined assessment time points at 5 min, 10 min, and 15 min after awakening (10). Secondary endpoints included the time to eye opening on verbal command (defined as the time interval from cessation of anesthesia to eye opening in response to verbal command) and the time to full awakening (defined as the time interval from cessation of anesthesia to full awakening). Concurrently, the non-invasive mean arterial pressure (MAP), heart rate (HR), respiratory rate (RR), and SpO_2_ were recorded at the following time points: T1 (upon entry into the operating room), T2 (after anesthetic stabilization and before the start of surgery), T3 (post-awakening after responding to verbal commands), and T4 (at the time of discharge from the operating room). Additionally, the incidence of adverse events during the surgery, such as motor response, assisted ventilation, and rescue analgesic use, was recorded.

### Study participants

The inclusion criteria for this study were as follows: (1) Patients must be aged 18–65 years (inclusive); (2) Patients should be scheduled to undergo elective hysteroscopic polypectomy under intravenous general anesthesia; (3) Patients must be classified as American society of anesthesiologists (ASA) physical status I-II; (4) The Body Mass Index (BMI) of patients must range from 18 kg/m^2^ to 30 kg/m^2^; (5) Patients had to demonstrate a thorough understanding of the purpose, content, procedures, and potential risks associated with the trial and voluntarily provide written informed consent.

The exclusion criteria for this study were as follows: (1) Patients who are unable to understand the Clinical Recovery Score (CRS) upon awakening and cooperate during the evaluation; (2) Patients with a history of chronic pain and long-term use of analgesics; (3) Patients with severe cardiovascular diseases; (4) Patients who are allergic to oliceridine, fentanyl, or sufentanil; (5) Patients who voluntarily withdraw from the study.

### Sample size

The primary endpoint of this study was the CRS measured 15 min after awakening. Based on previous studies (11), we anticipate an effect size (Cohen's f) of 0.35, which corresponds to an η^2^ of approximately 0.11. The significance level (α) is set at 0.05 (two-sided), and the desired statistical power (1-β) is 0.80. Using R software for calculations, we determined that the total required sample size is 84 participants. Taking into account a potential dropout rate of approximately 10%, we plan to recruit a total of 93 patients.

### Statistical analysis

Statistical analyses were performed using R software (version 4.5.0). Continuous variables are presented as mean ± SD or median (IQR), and categorical variables as counts and percentages. Baseline comparisons were performed using one-way ANOVA, the Kruskal–Wallis test, or Pearson's chi-square test, as appropriate. The primary endpoint, CRS, was analyzed as an ordinal repeated outcome using nonparametric repeated-measures analysis (nparLD), followed by Dunn's *post hoc* test with Bonferroni correction. Secondary recovery outcomes, including time to eye opening on verbal command and time to full awakening, were compared using the Kruskal–Wallis test with Dunn's *post hoc* test and Bonferroni correction, whereas intraoperative propofol dosage was compared using one-way ANOVA. Repeated physiological variables (HR, RR, MAP, and SpO_2_) were analyzed using linear mixed-effects models, followed by Tukey-adjusted pairwise comparisons. Intraoperative adverse events, including motor response, assisted ventilation, and rescue analgesic use, were compared using Pearson's chi-square test. A two-sided *p*-value < 0.05 was considered statistically significant.

## Results

**Figure 1:**
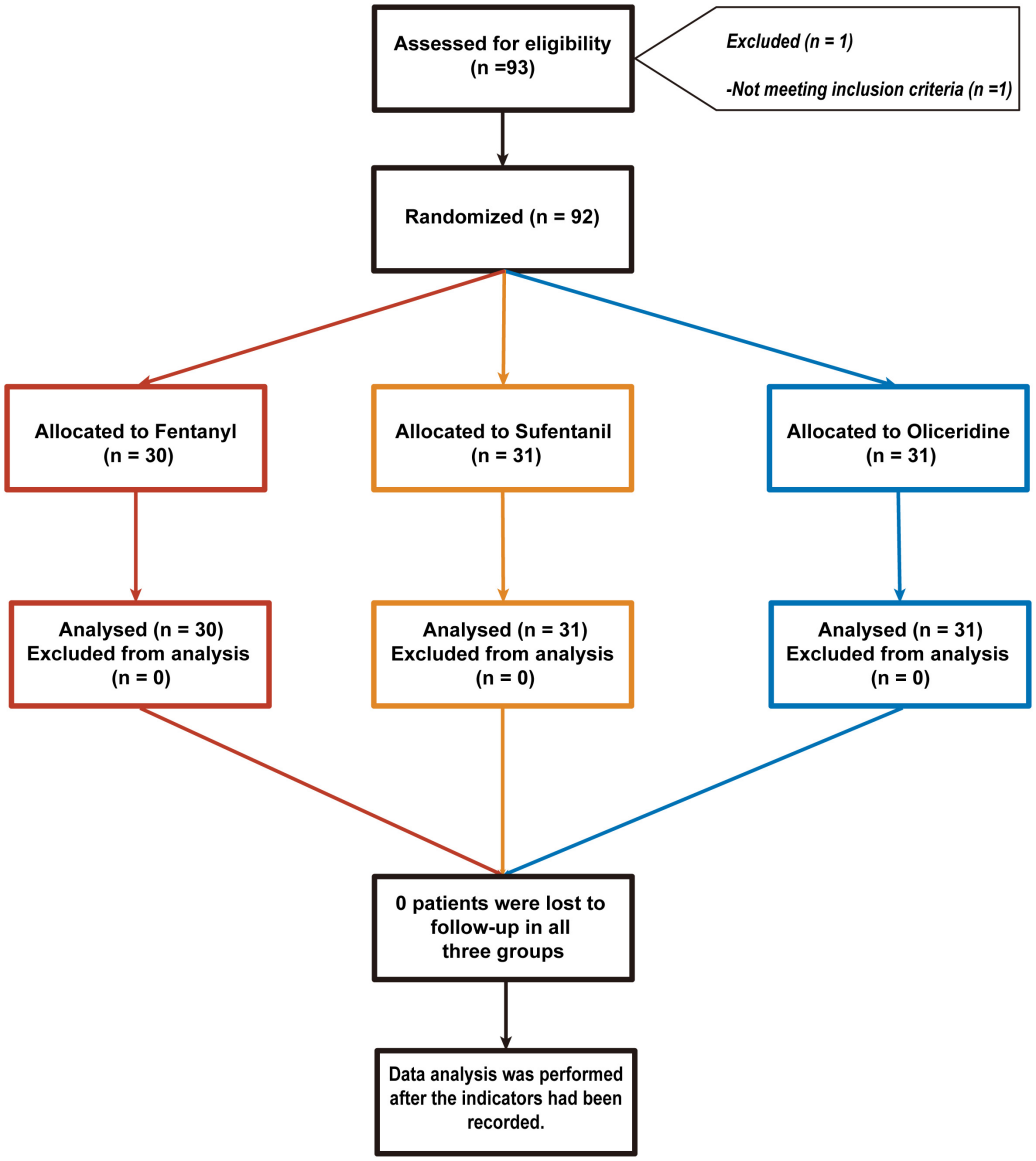
Consolidated Standards of Reporting Trials (CONSORT) flow diagram defining patient assessment and enrollment numbers in the present study.

Of the 93 patients enrolled in the study, one patient did not meet the inclusion criteria. Consequently, 92 patients were ultimately included in the analysis. These patients were divided into the following groups: fentanyl group (*n* = 30), sufentanil group (*n* = 31), and oliceridine group (*n* = 31), as shown in Figure 1. The mean age of the study subjects was 41 years (standard deviation [SD] = 11), and the mean body mass index (BMI) was 23.58 kg/m^2^ (SD = 3.08). All patients had an American Society of Anesthesiologists (ASA) physical status of Grade 1 (42%) or Grade 2 (58%). The mean operative time was 28.85 min (SD = 9.3), as presented in Supplementary Table S1. There were no differences between the groups in terms of basic demographic characteristics and surgical duration (Table 1).

**Table 1:** Patient characteristics.

Characteristic	Fentanyl	Sufentanil	Oliceridine	*p-value*
Age (yr)	40.63 ± 8.63	39.58 ± 10.55	43.45 ± 12.41	0.34
Height (cm)	158.87 ± 5.51	159.29 ± 5.66	157.16 ± 5.10	0.27
Weight (kg)	58.23 ± 9.14	60.00 ± 8.71	59.61 ± 8.48	0.71
BMI (kg/m^2^)	23.01 ± 3.17	23.60 ± 2.97	24.11 ± 3.09	0.39
Surgery duration (min)	29.07 ± 9.48	27.71 ± 10.32	29.77 ± 8.18	0.68

### The primary endpoint

Nonparametric repeated-measures analysis demonstrated significant main effects of group and time on CRS, whereas the group-by-time interaction was not significant (ANOVA-type statistic [ATS] *p* = 2.97 × 10^−5^ for group, *p* = 4.85 × 10^−112^ for time, and *p* = 0.865 for the group-by-time interaction). At 5 min post-awakening, overall between-group differences were significant (Kruskal-Wallis χ^2^ = 12.7, *p* = 0.002; ε^2^ = 0.120), and *post hoc* Dunn-Bonferroni comparisons showed higher CRS in the oliceridine group than in the fentanyl group (adjusted *p* = 0.001) and the sufentanil group (adjusted *p* = 0.008). Similar findings were observed at 10 min (χ^2^ = 9.52, *p* = 0.009; ε^2^ = 0.084) and 15 min (χ^2^ = 16.7, *p* < 0.001; ε^2^ = 0.165), with no significant difference between the fentanyl and sufentanil groups at any time point. Detailed median (IQR) values and pairwise effect estimates are presented in Table 2.

**Table 2:** Differences in clinical recovery score (CRS); data are median (IQR).

Time point	Fentanyl	Sufentanil	Oliceridine	*p-value*	Effect size (ε^2^ )
5 min	6 [5–7]	6 [6–7]	7 [6–8]^a, b^	0.002	0.120
10 min	8 [7–8]	8 [7–9]	8 [8–10]^a, b^	0.009	0.084
15 min	9 [8–10]	9 [8–10]	10 [9–11]^a, b^	< 0.001	0.165

^a^p < 0.05 compared with fentanyl group; ^b^p < 0.05 compared with sufentanil group.

### The secondary endpoints

Patients in the oliceridine group exhibited a shorter time to eye opening on verbal command than those in the fentanyl group (Cliff's delta = −0.56; 95% CI, −0.75 to −0.33) and the sufentanil group (Cliff's delta = −0.69; 95% CI, −0.86 to −0.49). Similarly, the oliceridine group had a shorter time to full awakening than the fentanyl group (Cliff's delta = −0.53; 95% CI, −0.75 to −0.28) and the sufentanil group (Cliff's delta = −0.58; 95% CI, −0.78 to −0.35). No significant difference was observed in intraoperative propofol dosage among the three groups (*p* = 0.646); the mean difference was 6.12 mg (95% CI, −32.01–44.25) for oliceridine vs. fentanyl and 16.45 mg (95% CI, −16.53–49.44) for oliceridine vs. sufentanil.

Detailed results are shown in Table 3.

**Table 3:** Propofol dosage, time to eye opening on verbal command, and time to full awakening.

Outcome	Fentanyl (*n* = 30)	Sufentanil (*n* = 31)	Oliceridine (*n* = 31)	*p-value*	Oliceridine vs. Fentanyl (Effect estimate [95% CI])	Oliceridine vs. Sufentanil (Effect estimate [95% CI])
Propofol dosage (mg)	240.33 ± 79.07	246.45 ± 94.39	246.45 ± 69.16	0.646	6.12 (-32.01, 44.25)	16.45 (-16.53, 49.44)
Time to eye opening on verbal command (min)	4.5 [3–6]	5 [4–6]	3 [3–3]^a, b^	< 0.001	−0.56 (-0.76,−0.33)	−0.69 (-0.86,−0.49)
Time to full awakening (min)	8 [7–9]	9 [7–11]	5 [5–7]^a, b^	< 0.001	−0.53 (-0.75,−0.28)	−0.58 (-0.78,−0.35)

Data are presented as mean ± SD for propofol dosage and median [IQR] for awakening-related outcomes. Pairwise effect estimates are reported as mean differences with 95% confidence intervals for propofol dosage and Cliff's delta with 95% confidence intervals for awakening-related outcomes. Negative Cliff's delta values indicate shorter times in the oliceridine group relative to the comparator group.

^a^p < 0.05 compared with fentanyl group.

^b^p < 0.05 compared with sufentanil group.

**Figure 2:**
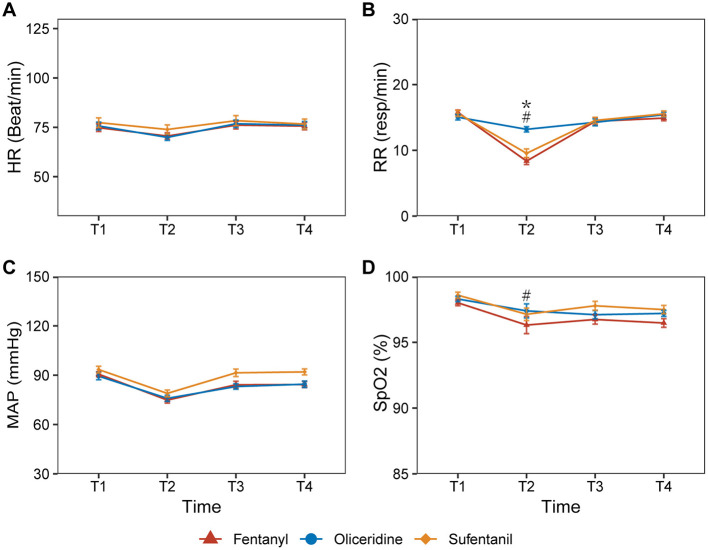
Changes in vital signs at predefined perioperative time points. **(A)** HR, heart rate; **(B)** RR, respiratory rate; **(C)** MAP, mean arterial pressure; **(D)** SpO_2_, pulse oxygen saturation. T1, upon entry into the operating room; T2, after anesthetic stabilization and before surgery; T3, post-awakening after response to verbal command; T4, at operating room discharge. *oliceridine group compared with sufentanil group (*p* < 0.001); ^#^oliceridine group compared with fentanyl group (*p* < 0.05). Data are presented as mean ± SD. Detailed between-group comparisons at each time point are provided in Supplementary Table S1.

At T2, the oliceridine group had higher RR than both the fentanyl group (13.19 ± 2.24 vs. 8.37 ± 2.86 resp/min) and the sufentanil group (13.19 ± 2.24 vs. 9.55 ± 3.70 resp/min). At the same time point, SpO_2_ was higher in the oliceridine group than in the fentanyl group (97.39 ± 2.96 vs. 95.87 ± 4.21%), whereas no significant difference was observed between the oliceridine and sufentanil groups (97.39 ± 2.96 vs. 96.87 ± 2.66%). No significant between-group differences in RR were observed at T1, T3, or T4. At T3 and T4, MAP was higher in the sufentanil group than in both the fentanyl group (91.45 ± 12.65 vs. 84.17 ± 11.56 mmHg, *p* = 0.033, at T3; 92.00 ± 10.32 vs. 84.30 ± 10.20 mmHg, *p* = 0.023, at T4) and the oliceridine group (91.45 ± 12.65 vs. 80.84 ± 15.37 mmHg, *p* < 0.001, at T3; 92.00 ± 10.32 vs. 84.52 ± 10.81 mmHg, *p* = 0.026, at T4), while no significant difference was observed between the fentanyl and oliceridine groups. HR remained comparable across the three groups. These results are presented in Figure 2, with detailed data provided in Supplementary Table S1.

Regarding adverse events, no significant differences were observed in the incidence rates of intraoperative motor responses, the need for assisted ventilation, or the utilization of rescue analgesics among the three patient groups. The results are detailed in Table 4.

**Table 4:** Report of intraoperative adverse events.

Outcome	Fentanyl (*n* = 30)	Sufentanil (*n* = 31)	Oliceridine (*n* = 31)	*p-value*
Motor response	13 (43.33%)	9 (29.03%)	9 (29.03%)	0.40
Assisted Ventilation	10 (33.33%)	6 (19.35%)	3 (9.68%)	0.14
Rescue analgesic use	14 (46.67%)	10 (32.26%)	9 (29.03%)	0.31

## Discussion

Compared with fentanyl and sufentanil, oliceridine was associated with improved early recovery profiles and more favorable respiratory-related observations in patients undergoing hysteroscopic polypectomy. Specifically, patients receiving oliceridine demonstrated shorter awakening times and higher CRS scores, along with higher RR at T2 and higher SpO_2_ than fentanyl at the same time point. These findings suggest potential differences in perioperative recovery characteristics among the three regimens. Although the absolute differences in CRS between groups were numerically modest, they were accompanied by shorter awakening times and consistent directional differences across multiple early recovery outcomes, suggesting a broader pattern of improved immediate post-anesthetic recovery rather than an isolated score difference.

In the present study, RR at T2 was higher in the oliceridine group than in the fentanyl and sufentanil groups. At the same time point, SpO_2_ was higher in the oliceridine group than in the fentanyl group, whereas no significant difference was observed between the oliceridine and sufentanil groups. While these findings may suggest differences in early respiratory patterns, RR and SpO_2_ remain indirect measures that can be influenced by multiple factors, including anesthetic depth and nociceptive stimulation. Therefore, these observations should be interpreted cautiously and do not, by themselves, establish reduced respiratory depression.

Regarding hemodynamic parameters, MAP at T3 and T4 was higher in the sufentanil group than in both the fentanyl and oliceridine groups, whereas no significant difference was observed between the fentanyl and oliceridine groups at these time points. HR was comparable across all groups. These findings indicate that fentanyl and oliceridine had similar MAP levels at T3 and T4, while the sufentanil group maintained higher MAP values at these time points.

Oliceridine is a G protein-biased μ-opioid receptor agonist, a pharmacological property that has been proposed to differentially modulate analgesic and adverse effect pathways in preclinical studies. Early investigations in β-arrestin2 knockout models suggested enhanced analgesic responses and reduced opioid-related adverse effects, which contributed to the development of biased agonism as a research concept (12). Subsequent studies using similar experimental models have provided additional support for this concept (13, 14). Preliminary studies have suggested that, compared with conventional opioids such as morphine, oliceridine may be associated with a lower incidence of respiratory and gastrointestinal adverse effects in preclinical models and early-phase clinical settings (15). In addition, clinical studies in surgical populations have reported a low incidence of respiratory safety events in patients receiving oliceridine for postoperative analgesia (16, 17). Within ERAS protocols, mitigating opioid-related side effects while accelerating emergence is crucial for facilitating early mobilization and reducing postoperative nausea. Future research should validate whether these intraoperative advantages translate into improved long-term outcomes, thereby solidifying the role of Oliceridine in modern rapid-recovery anesthetic strategies.

However, these findings arise from heterogeneous study designs and populations, and direct comparisons should be made with caution. In the context of the present study, the observed differences in recovery and respiratory-related parameters are broadly consistent with these prior reports but do not provide mechanistic confirmation.

Our findings indicated that there was no statistically significant difference in the intraoperative propofol dosage administered among the three patient groups (*p* > 0.05). Although BIS monitoring was used, equivalent propofol dosing does not necessarily ensure identical anesthetic depth throughout the procedure, and the potential influence of anesthetic depth on recovery outcomes cannot be fully excluded. GPCR signaling is generally considered central to opioid analgesic effects, whereas β-arrestin2 has been implicated in several opioid-related adverse effects (18–20). As a member of the arrestin family, β-arrestin2 functions as a multifunctional adaptor protein involved in receptor desensitization, endocytic trafficking, and intracellular signaling pathways (21–23). Although the observed differences in recovery profiles may be contextualized within the framework of biased μ-opioid receptor signaling, the present study did not include mechanistic or biomarker assessments. Therefore, such interpretations remain speculative and should be considered hypothesis-generating rather than confirmatory.

However, this study has several limitations. First, it was a single-center study with a relatively small sample size, which may limit the generalizability of the findings. Second, although treatment allocation was masked, postoperative CRS assessment was performed by the attending anesthesiologist rather than by an independent blinded assessor; therefore, observer-related assessment bias may not be completely excluded. Third, the assessment indicators for recovery quality still warrant further refinement. Although CRS was used as a structured measure of early postoperative recovery, it may not fully capture all dimensions of modern recovery assessment. Future studies should incorporate multicenter, large-sample investigations to further evaluate the efficacy and safety of oliceridine in hysteroscopic surgery, particularly in elderly and high-risk populations. In addition, the potential risk of perioperative complications requires further investigation.

In summary, in patients undergoing hysteroscopic polypectomy under non-intubated general anesthesia with propofol, the use of oliceridine was associated with improved early recovery outcomes and more favorable respiratory-related observations compared with fentanyl and sufentanil. These findings support the potential value of oliceridine in promoting earlier recovery in day surgery, although larger studies with more comprehensive physiological monitoring are needed to confirm its respiratory safety profile.

## Data Availability

The original contributions presented in the study are included in the article/supplementary material, further inquiries can be directed to the corresponding authors.
