# Supplementary material for: Efficacy and safety of oliceridine in daytime hysteroscopic polypectomy: a randomized, double-blind, single-center controlled trial
Source: Front Med (Lausanne). 2026 May 22;13:1819353. doi: 10.3389/fmed.2026.1819353 (PMC13237686; doi:10.3389/fmed.2026.1819353)
Supplement: Supplementary file 1 [file Data_Sheet_1.pdf]

Table S1 Vital signs at each time point

	Group	T1	T2	T3	T4
**MAP (mmHg)**	**Fentanyl**	90.60 ± 7.98	74.87 ± 10.03	84.17 ± 11.56	84.30 ± 10.20
	**Sufentanil**	93.42 ± 11.09	78.94 ± 11.49	91.45 ± 12.65	92.00 ± 10.32
	**Oliceridine**	89.42 ± 12.32	75.90 ± 10.20	80.84 ± 15.37	84.52 ± 10.81
**SpO_2** (%)	**Fentanyl**	98.00 ± 1.20	95.87 ± 4.21	96.73 ± 1.95	96.47 ± 1.80
	**Sufentanil**	98.58 ± 1.29	97.13 ± 2.66	97.77 ± 1.89	97.48 ± 1.77
	**Oliceridine**	98.29 ± 1.13	97.39 ± 2.96	97.10 ± 1.74	97.19 ± 1.30
**HR (beats/min)**	**Fentanyl**	74.80 ± 10.53	70.57 ± 9.06	76.10 ± 10.54	75.63 ± 10.11
	**Sufentanil**	77.39 ± 13.39	73.90 ± 12.81	78.35 ± 14.21	76.65 ± 14.12
	**Oliceridine**	75.68 ± 10.80	69.90 ± 8.61	74.71 ± 15.46	76.06 ± 10.37
**RR (Resp/min)**	**Fentanyl**	15.77 ± 1.96	8.37 ± 2.86	14.40 ± 3.10	14.90 ± 2.22
	**Sufentanil**	15.61 ± 2.58	9.55 ± 3.70	14.55 ± 2.57	15.55 ± 2.43
	**Oliceridine**	15.03 ± 2.32	13.19 ± 2.24	14.26 ± 2.94	15.42 ± 2.08

MAP : Mean Arterial Pressure; **SpO_2**: Pulse Oxygen Saturation; **RR**: Respiratory Rate; **HR**: Heart Rate

Data are presented as mean ± SD. **T1**, upon entry into the operating room; **T2**, after anesthetic stabilization and before the start of surgery; **T3**, post-awakening after responding to verbal commands; **T4**, at the time of discharge from the operating room. Pairwise between-group comparisons at each time point were performed using linear mixed-effects models with Tukey-adjusted post hoc tests. Significant differences were observed for RR at T2 (oliceridine vs fentanyl and sufentanil), for SpO_2 at T2 (oliceridine vs fentanyl), and for MAP at T3 and T4 (sufentanil vs fentanyl and oliceridine). No significant between-group differences were observed for HR.
